# The Extracellular Matrix: A Key Accomplice of Cancer Stem Cell Migration, Metastasis Formation, and Drug Resistance in PDAC

**DOI:** 10.3390/cancers14163998

**Published:** 2022-08-18

**Authors:** Dan Wang, Yuqiang Li, Heming Ge, Tarik Ghadban, Matthias Reeh, Cenap Güngör

**Affiliations:** Department of General Visceral and Thoracic Surgery, University Medical Center Hamburg-Eppendorf, 20251 Hamburg, Germany

**Keywords:** pancreatic ductal adenocarcinoma, extracellular matrix, cancer stem cells, chemotherapy resistance, metastasis

## Abstract

**Simple Summary:**

This review takes the extracellular matrix (ECM) as the starting point, and describes its influence and related mechanisms on the biological behavior of pancreatic ductal carcinoma (PDAC). It focuses on how the ECM regulates cancer stem cells and thus affects the metastasis and drug resistance of PDAC. Finally, current and ongoing treatment strategies for ECM are presented. ECM-related factors and mechanisms are both the focus and difficulty of current therapeutic strategy research, but further exploration is crucial for effectively eradicating the disease and improving patient survival.

**Abstract:**

Pancreatic ductal adenocarcinoma (PDAC) is rich in dense fibrotic stroma that are composed of extracellular matrix (ECM) proteins. A disruption of the balance between ECM synthesis and secretion and the altered expression of matrix remodeling enzymes lead to abnormal ECM dynamics in PDAC. This pathological ECM promotes cancer growth, survival, invasion, and alters the behavior of fibroblasts and immune cells leading to metastasis formation and chemotherapy resistance, which contribute to the high lethality of PDAC. Additionally, recent evidence highlights that ECM, as a major structural component of the tumor microenvironment, is a highly dynamic structure in which ECM proteins establish a physical and biochemical niche for cancer stem cells (CSCs). CSCs are characterized by self-renewal, tumor initiation, and resistance to chemotherapeutics. In this review, we will discuss the effects of the ECM on tumor biological behavior and its molecular impact on the fundamental signaling pathways in PDAC. We will also provide an overview of how the different ECM components are able to modulate CSCs properties and finally discuss the current and ongoing therapeutic strategies targeting the ECM. Given the many challenges facing current targeted therapies for PDAC, a better understanding of molecular events involving the interplay of ECM and CSC will be key in identifying more effective therapeutic strategies to eliminate CSCs and ultimately to improve survival in patients that are suffering from this deadly disease.

## 1. Introduction

Pancreatic ductal adenocarcinoma (PDAC) is one of the most aggressive and lethal cancers in the world, with a dismal overall 5-year survival rate of about 9% [1]. Some studies predict PDAC will be on track to develop into the second leading cause of cancer death by 2030 [2,3]. Unfortunately, a large proportion of patients have locally advanced stages or regional/distant metastatic spread at the time of initial diagnosis, making the majority of patients not suitable for curative surgery [4]. Although great progress has been made in the treatment of PDAC, such as chemotherapy, radiotherapy, and molecular targeting, it is still challenging that many patients show intrinsically or acquired resistance to these treatments, and the therapeutic effect and benefit is often limited [5,6]. Although neglected for many years, much attention has been paid to investigate the local tumor microenvironment. It has been found that the abnormal dynamics of the extracellular matrix (ECM) is a major promoting factor for early metastasis and drug resistance of PDAC cells [7,8].

The ECM consists of numerous structural proteins such as collagen, proteoglycan and glycoproteins, and stromal cell proteins (non-structural proteins) [9]. In principle, the ECM is not a static network scaffold, but regulates its biochemical function and biomechanical properties by dynamically regulating its composition, post-translational modifications, and structure [10]. Physiologically, mammalian cells and ECM are closely intermediated through various soluble factors such as cytokines, chemokines, costimulatory molecules, additional biological mediators (oxidants and prostaglandins), and physical stimuli such as microenvironmental stiffness and tension/compression forces [11]. The ECM represents a dynamic ecological niche in tumor development, with specific physical, biochemical, and biomechanical properties that help to maintain the homeostasis of cells, tissues, and organs [12]. Metastasis formation of invasive tumors is a complex and discrete series of various biological steps, but each step seems to be related to the dynamic regulation of ECM components and its post-translational modifications [13]. Changes in the ECM can directly induce cell transformation and metastasis to promote tumor initiation and progression [14]. A well-known clinical characteristic of PDAC is a strong desmoplastic reaction that is induced by cancer cells. This desmoplasia, defined as the growth of fibrous or connective tissue, is strongly associated with malignant neoplasms and causes a dense fibrosis around the tumor, tissue hypervascularization, and suppression of immune cells. This dense fibrotic matrix is interwoven with the ECM and is thought to provide a physical barrier protection for tumor cells. In addition, the deposition of large amounts of ECM proteins is common in solid tumors such as PDAC and is known as a pro-connective tissue hyperplasia response, which regulates stromal cell behavior, determines tumor-related inflammation and angiogenesis, and promotes a pro-tumor microenvironment [15].

Strikingly, cancer stem cells (CSCs) play an important role in the metastasis formation and chemotherapy resistance of PDAC [16]. Recent evidence suggests that ECM not only physically supports stem cells, but also directly or indirectly regulates the maintenance, proliferation, self-renewal, differentiation, and survival of stem cells (14). The ECM anchors stem cells to ecological niches where they maintain contact with guiding cues that regulate their existence. Furthermore, intracellular signaling pathways that influence cell fate and behavior can be triggered individually or jointly by the perception of mechanical forces that are generated by ECM, binding of ECM to cell surface receptors, and release of ECM-bound growth factors [17].

In summary, the cross-regulation and interaction network of ECM is crucial for metastasis and chemotherapy resistance of PDAC. The dynamic environment of ECM provides a reservoir for various oncogenic signaling molecules and supports the regulation of CSCs through mechanical forces and biochemical signals, thus promoting tumor cell metastasis and chemotherapy resistance.

In this review, we will summarize the current state of knowledge in ECM dynamics, its regulatory relationship with CSCs, and potentially new and ongoing of ECM targeting strategies in PDAC.

## 2. Composition and Role of the ECM in Health

The ECM is generally divided into two types: interstitial connective tissue matrix and basement membrane. The main components of interstitial ECM include fibrous proteins and proteoglycans, which play a role in maintaining tissue mechanical stiffness and hydration, respectively. The basement membrane is composed of a specific collagen network and laminin that not only provides structural support, but also binds several key growth factors and cytokines that regulate cell differentiation and maintain tissue homeostasis [18].

Collagen is the most abundant structural protein in the ECM, accounting for 30% of the total protein mass [19]. At present, 28 types of collagens that are encoded by 43 genes have been identified, all of which are composed of three polypeptide chains (α chains) that intertwine with each other in a rope-like fashion, forming a triple helix, and nucleating at the N-terminal [20]. The main ECM component of interstitial tissue is fibrous Type I collagen, which is a typical fibrous collagen. Type IV collagen is a non-fibrous collagen that is the main component of the basement membrane on which cells adhere and interact extensively with. Other non-fibrous collagen proteins, collagen XV and XVIII, are also expressed in the basement membrane. The functions of these scaffold proteins include enhancement of tissue tensile strength, guiding tissue development, regulating cell adhesion, and supporting chemotaxis and migration [19,21]. Collagen provides tissue strength and toughness by cross-linking molecules to form macromolecules [22]. Fibrous collagen influences many aspects of cell behavior by its physical properties, such as fiber size, organization, density, stiffness, and pore size between fibers, acting as ligands for integrin and non-integrin receptors, as well as reservoirs for growth factors and peptide mediators [23].

Elastin, which is another major ECM fiber, is abundant in ligaments and blood vessel walls. Elastin is highly elastic due to its amino composition and dynamic three-dimensional structure, maintaining tissue toughness and strength by resisting tissue deformation or rupture [24]. Although the amount of fibronectin in the ECM is small, it has many functions. Fibronectin is important for cell migration during development and plays a key role in mediating cell attachment. More importantly, fibronectin is involved in guiding the organization of interstitial ECM, linking various structural proteins in the ECM to form an integrated matrix [21,25]. Fibronectin can also exert regulatory functions through direct interactions with other proteins. Fibronectin, for example, contains an abundant arginine-glycine-asparagine (RGD) sequence that recognizes and binds to integrins on the cell membrane and has profound effects on intracellular signal transduction [26,27]. Laminin, which can participate in the formation of the basement membrane together with collagen, mainly plays an important role in the process of angiogenesis [28]. Laminin is composed of three different chains, an α chain, a β chain, and a γ chain, that are encoded by different genes. The α chain determines the specific biological activity and tissue distribution of each laminin [29]. Currently, studies have confirmed the presence of five forms of α chains (LAMA1-5) and three forms of β chains (LAMB1-3) and γ chains (LAMC1-3) in laminin [30]. Combinations of these different chains can produce more than 15 isoforms of laminin [29]. Laminin is distributed in a tissue-specific manner and plays different roles in each tissue. Laminin 511 (Lm511) and laminin 521 (Lm521) in the α5 laminin group are most widely distributed in the basement membranes of various types of epithelial cells and blood vessels [31]. Laminin 332 (Lm332) is a structurally and active laminin that is primarily organized in the basement membranes of skin and many other epithelial cells, which supports efficient cell adhesion and migration by binding to specific integrins (α3β1, α6β1, and α6β4) [32,33]. In addition, most of the extracellular stroma is filled with abundant proteoglycans in the form of hydration gels. Non-sulfated (Hyaluronic acid) and sulfated (chondroitin sulfate, heparin sulfate, and keratin sulfate) glycosaminoglycans bind to proteins through covalent bonds to form proteoglycans [34]. Due to its strong hydrophilic and highly extended conformation, it is conducive to the formation of hydrogel, so that the corresponding matrix can withstand high compression forces [35]. Traditionally, hyaluronic acid was mainly considered as a structural component in physiology, but some other functions have also been discovered. For example, hyaluronic acid can activate cell-to-cell contact-mediated signal transduction through CD44 or its mediated motor receptors [36,37].

ECM is a large family whose members include, but are not limited to, various matrix proteins, glycosaminoglycans, fibroblasts, growth factors, and specific enzymes [38]. Moreover, the ECM is a hyperactive structure whose equilibrium is critical for maintaining organizational homeostasis. This dynamic stability is maintained through a balance between metalloproteinases and tissue inhibitors of metalloproteinases, control of crosslinking enzyme activity, and ECM-bound growth factors [39]. The ECM not only affects cell fate and interacts with cells to regulate proliferation and differentiation, but also controls the host tissue response [40,41,42]. Cells that are in contact with the ECM sense the properties of the ECM through receptors and focal adhesion complexes in order to maintain tissue homeostasis. At the same time, cells in turn regulate the expression of ECM components and enzymes in response to signals from the ECM. This creates a feedback mechanism in which cells can also influence the ECM, leading to a balance between deposition and degradation of ECM components [43].

## 3. ECM in PDAC

Abnormal remodeling of the ECM is associated with a variety of pathologic conditions, such as inflammation, fibrosis, and cancer [40]. In particular, a growing number of studies have highlighted the active role of dysregulated ECM dynamics in tumor progression and invasion [18,44]. The expression of many ECM remodeling enzymes is frequently deregulated in human cancers, most notably Matrix metalloproteinases (MMPs) [45]. MMPs are secreted by cancer-associated fibroblasts (CAFs) in PDAC [46]. Fibroblasts in the tumor microenvironment, collectively referred to as CAFs, are associated with tumor aggressiveness and reduced survival [47,48]. The CAF population, due to the different cellular origins, exhibits marked heterogeneity and functional diversity [49]. One of the most common cellular origins of CAFs is pancreatic stellate cells (PSCs) [50]. After pancreatic injury, or TGF-β stimulation, PSCs were activated, resulting in changes in cell morphology from stellate to spindle, increased nuclear volume, and decreased vitamin A droplets [51,52,53]. CAFs can also originate from tissue-resident fibroblasts, which can be activated under the control of growth factors (such as TGF-β) [54]. Additional studies have demonstrated that CAFs may also arise from trans-differentiation of non-fibroblast lineages or epithelial cells, as well as the recruitment and differentiation of bone marrow-derived mesenchymal stem cells [46,55]. Markers that were used to distinguish different CAF subsets include α-SMA, platelet-derived growth factor receptors α and β (PDGFRα/β), FAP, and Ca^2+^ binding protein (S100A4), but none of them are exclusively expressed by CAFs [55,56]. In terms of functional diversity, CAFs can secrete MMP, which promotes the degradation of the ECM and the release of various factors, and leads to the recruitment of some specific cells and various cytokines. On the other hand, CAFs are mainly responsible for the deposition of dense tumor stroma, which can serve as a structural scaffold for cell interactions and a physical barrier against immune infiltration. Furthermore, many growth factors and proinflammatory cytokines, including interleukin 6 (IL-6), TGF-β, and vascular endothelial growth factor (VEGF) can be produced by CAFs, which can promote tumor growth, angiogenesis, and assists in immune escape [46,57]. Therefore, CAFs, combining the above characteristics, are often programmed into three different subgroups: “antigen-presenting”, “myofibroblastic”, and “inflammatory” CAFs [58].

The dysregulation of ECM remodeling can cause mutant cells to escape apoptosis due to the pro- and anti-apoptotic effects of various ECM components or their functional fragments [59]. Abnormal ECM remodeling affects the behavior of not only cancer cells, but also those of stromal, endothelial, and immune cells of the local microenvironment as well [60,61]. Angiogenesis that is induced by abnormal ECM remodeling often results in an abnormal vasculature that is characterized by tortuous and immature leaky vessels in malignancies [62]. Vascular abnormalities, including the absence of a lymphatic network within the tumor, lead to increased interstitial fluid pressure, acidosis, and hypoxia, which underlie metastasis and drug resistance [63]. IL-6 that is secreted by stromal CAFs recruits tumor-associated macrophages and promotes their transition to an immunosuppressive phenotype (M2) [64]. Stromal CAFs recruit and induce Treg differentiation to suppress antitumor immunity and similarly enhance the tumor-promoting function of CD4+ helper T (Th) lymphocytes [65]. Moreover, the dense fibrotic ECM that is induced by CAFs can also physically prevent immune cells from effectively infiltrating tumors, greatly limiting T-cell contact with cancer cells [66].

A prominent feature of PDAC is a strong pro-fibrotic response leading to a hyper-fibrotic stroma. Sparse vascularization and abundant deposition of extracellular components lay the groundwork for the stroma of PDAC [67]. Collagen, produced mainly by CAFs (especially derived from PSCs), is the most representative component of PDAC connective tissue [68]. Fibrinous collagen Type I and III account for more than 90% of the total and protein levels of these collagen proteins that increased nearly three-fold during the progression of PDAC [69]. However, compared with normal tissue, the ratio of these two kinds of collagen in tumor connective tissue did not change significantly, so one should pay attention to other differentially expressed collagen in the process of PDAC [70]. Other collagen proteins, including types IV, V, VI, VII, XII, XIV, and XV, are also key participants in PDAC tumorigenesis and can play both beneficial and harmful roles. Collagen IV, produced by cancer cells, helps cancer cells to proliferate, migrate, and reduce apoptosis. Therefore, the high level of Type IV collagen in patients after surgery often indicates the possibility of recurrence and low survival rates [71,72]. Moreover, ECM collagen interacts with integrins (α- and β-subunits), expressed on the surface of PDAC cells to promote the proliferation and migration of tumor cells [73,74]. Conversely, the overexpression of Type XV collagen during pancreatic tumorigenesis is detrimental to the ability of cancer cells to migrate into the matrix, which is rich in Type I collagen [75]. Surprisingly, in addition to directly promoting tumor progression and signaling, collagen can also serve as a nutritional source for tumor cells. PDAC cells can metabolize collagen molecules and produce proline that enables cancer cell proliferation in the special hypoxic and low-nutrient environment of tumors [76].

Proteoglycans and glycoproteins are composed of core proteins that undergo post-translational glycosylation, which largely shapes their conformation and cell signaling functions, and has important effects on tumor cells [77]. Most subunits of laminin, a small leucine-rich proteoglycan, are overexpressed in PDAC and are related to poor prognosis [78]. In particular, Lm332 and Lm511/521, which are currently common in various tumors, promote tumor progression by interacting with other proteins and cytokines [33]. The interaction of Lm332 with collagen Type VII and integrin α3β1 promotes tumor cell growth and metastasis, respectively [79,80]. Fibronectin has been shown to act as scaffolds and regulate cellular processes in the PDAC microenvironment [81]. Fibronectin stimulates tumor cell proliferation and invasion of the basement membrane and acts as a bridging molecule between ECM collagen and integrin [82,83]. Moreover, studies have shown that reduced galectin-1 (GAL1) in mouse models leads to reduced matrix activation and increased cytotoxic T-cell infiltration [84]. GAL1, as a class of glycoproteins, is expressed in multiple tumor types and is involved in proliferation, invasion, angiogenesis, metastasis, and is associated with patient survival [85,86]. The expression of GAL1 is upregulated in the PDAC tumor microenvironment and is low in long-term (≥10 years) survivors of PDAC [85,87]. The transforming growth factor β1 (TGF-β1) protein regulates cell adhesion through various integrins (αvβ3, αvβ5, and α1β1). TGF-β1 is increased in PDAC tissues and inhibits tumor cells by reducing proliferation and activation of tumor CD8+ T-cells or by promoting their migrative and invasive properties [88,89]. A previous study has confirmed that testican is a proteoglycan that promotes tumor resistance through mediated epithelial-mesenchymal transition signaling [90]. Lumican is a small leucine-rich proteoglycan that directly binds and inhibits MMP14, thereby preventing extracellular matrix collagen hydrolysis by this enzyme [91]. Therefore, testican promotes the growth and invasion of PDAC cells by influencing collagen deposition, while lumican interferes with tumor progression and prolongates the survival of patients by restricting the growth and metastasis of cancer cells [92,93]. Versican is an extracellular matrix proteoglycan that plays key roles in tumor cell invasion, metastasis, and angiogenesis [94]. In addition, versican is an immunosuppressive component that is detrimental to patient survival by reducing T-cell infiltration, while core proteoglycan is an anti-tumor component that slows down tumor cell growth [69,95]. The interaction of these proteoglycans with hyaluronic acid regulates the hydration level of the interstitial fluid and thus interstitial pressure.

Compared with normal pancreas, the total glycosaminoglycan content was significantly increased in PDAC (four-fold, *p* ≤ 0.001), mainly due to increased hyaluronic acid content [96]. A large amount of hyaluronic acid is already produced in the tumor microenvironment in the pre-cancerous intraepithelial neoplasia (PanIN) stage [97]. Hyaluronic acid is thought to be deposited primarily by CAFs and to some extent by PDAC cells [98,99]. Most importantly, hyaluronidase breaks down the hyaluronic acid matrix to allow interactions between growth factors and growth factor receptors, promoting glucose metabolism, tumor cell proliferation, and migration [100].

PSCs are key to maintaining the balance between ECM synthesis and degradation, and most studies showed an “activated” state in PDAC, leading to excessive deposition of ECM proteins [101]. It also leads to the increased expression of collagen, αSMA, immunomodulatory and other pro-tumorigenic genes [101]. TGF-β promotes ECM deposition and tumor progression in late stages of carcinogenesis [102]. As a potent activator of PSC, TGF-β mediates the interaction between the tumor microenvironment and tumor cells by binding to TGF-β cell surface receptors, promoting the deposition of ECM proteins including fibronectin and collagen [103]. The PSCs, once activated, further modulates ECM through various mechanisms. Previous studies have suggested that tissue stiffness can enhance the proliferation of tumor cells [104]. Collagen cross-linking by Lysyl-oxidase (LOX) and Tissue-transglutaminase 2 (TG2) enhances matrix stiffening [105,106]. Driven by TG2 and/or LOX, additional cross-linked collagen and rigid ECM activate Yes-associated protein (YAP) and the transcriptional coactivators of PDZ-binding motifs (TAZ), which enhances cell proliferation [107]. The stiffened ECM matrix in turn regulates the activity of ECM MMPs, Vimentin, and E-cadherin [107,108].

## 4. ECM and Pancreatic Cancer Stem Cells (PCSCs)

The tumorigenic ability of each PDAC cell is heterogenous, and the development and proliferation of PDAC are highly dependent on the restricted PDAC cell subpopulation, namely pancreatic cancer stem cells (PCSCs) [109]. The activity of stem cells is dependent on exogenous niche factors in normal tissues and organs. Similarly, significant changes within the tumor microenvironment in PDAC can have a similar effect on PCSCs. The tumor microenvironment of PDAC strongly promotes fibroplasia, in which the ECM interacts with integrins (including integrin subunits β1, α6, and β3) to regulate PCSCs function by influencing autocrine and paracrine signaling pathways [110]. Type 1 collagen is the main scaffold for CD133-positive CSCs and promotes cell invasion through the PI3K/AKT pathway and also increases PCSCs enrichment by activating Focal Adhesion Kinase (FAK) [111]. Abnormal collagen cross-linking creates mechanical stresses that increase the rigidity and stiffness of the cancer matrix, which are important factors that are transmitted to CSC and regulate its proliferation and plasticity [112]. The ECM can also dynamically influence CSC ecology by creating a hypoxic environment [113]. On the one hand, hypoxia can regulate the “niche” of PCSCs by directly activating Hypoxia-inducible factor (HIF) and its target genes [114]. On the other hand, HIF can also regulate signaling pathways and transcription factors that are critical for maintaining stem cell self-renewal and pluripotency, such as Oct-4 and Notch signaling cascades [115]. Furthermore, the high expression of HIF-1α under hypoxia can enhance the expression of PCSC markers, chemotherapeutic resistance, and an epithelial-mesenchymal transformation (EMT) phenotype [116].

Nevertheless, proteoglycans bind to various cytokines and chemokines in the tumor microenvironment to activate multiple signaling pathways in CSCs, including Notch, Wnt, and Hedgehog [117]. Transcriptional activation, mediated by the Wnt/β-catenin signaling pathway, induces the expression of C-MYC and SOX2, thereby promoting cancer stemness [118]. Glypican-4 (GPC4), as a typical proteoglycan, is associated with various human malignancies [119,120]. A study revealed a role for GPC4 in PCSC stemness regulation and the inhibition of GPC4 attenuates the stem cell-like properties by inhibiting the Wnt/β-catenin pathway [121]. Syndecan-1, a well-known multifunctional integrator of cell surface signaling, regulates components of STAT3/NF-κB signaling and connects them with the AKT pathway in a Notch-activation-dependent manner to promote stemness-related phenotypic traits [122].

In addition, hyaluronic acid is a major ECM component of the stem cell niche, is frequently overexpressed in PDAC, and affects stromal cell behavior, which provides a favorable microenvironment for CSC self-renewal and maintenance [123]. This process mainly relies on the interaction of hyaluronic acid with its main receptor CD44. The hyaluronic acid-CD44 axis modulates the stemness properties of CSCs by inducing the EMT program and the secretion of extracellular vesicles. Specifically, excessive hyaluronic acid can activate AKT and ERK1/2 to induce EMT, and CD44 was found to be an important factor in TGF-β-induced EMT [124]. Intracellular hyaluronic acid-induced EMT increases centrosome abnormalities and micronucleation, creating a suitable niche for CSCs [125]. Notably, CSC-derived extracellular vesicles regulate communication between CSCs and their niche [126]. Studies have confirmed that tumor-initiating cells (CICs)-derived exosomes with CD44v6 in PDAC can transfer migration and invasion capabilities to non-CICs, and regulate the expression of integrins and proteases to promote cancer cell migration and invasion. Once CD44v6 was knocked down, it altered the composition of secreted tumor exosomes and lost the ability to promote the malignant phenotype. This indicates that the induction of the pre-metastatic niche by exosomes is dependent on CD44v6, further demonstrating the importance of the hyaluronic acid-CD44 interaction in extracellular vesicles promoting malignancy [127].

## 5. ECM, PCSCs, and Metastasis

PCSCs, one of the main forces that are responsible for cancer metastasis, have the ability to evade treatment, exosmosis, and colonization from the primary tumor site to the site of secondary metastasis [128]. The niche formation of the metastatic site drives the transfer of CSCs [7]. Interactions between CSCs and the microenvironment such as CAFs and immune cells, as well as with the cell matrix, contribute to CSC migration and the priming of metastatic sites [7]. Another major factor in the formation of metastatic niches is the presence of extracellular vesicles, which are exosomes that also contain ECM regulatory genes [112]. The cross-linking of collagen and elastin in the ECM is primarily regulated by the LOX protein family, which consists of LOX-1 and four related enzymes, LOX -like protein (LOXL1-4) [129,130,131]. LOXL2, in particular, leads to the stiffening of PDAC tissues by promoting the cross-linking of collagen fibers, and increases the secretion of related factors (such as exosomes) in primary tumor tissues, which leads to ECM remodeling or stromal cell recruitment, and promotes the formation of an ecological niche before secondary organ metastasis, and ultimately promotes metastatic formation [132]. Furthermore, the overexpression of LOXL2 in mouse models increases EMT and stemness, thereby promoting primary and metastatic tumor growth and reducing the overall survival [132].

Some specific ECM molecules, including hyaluronic acid and Tenascin-C, have become the focus of research on the mechanism of tumor metastasis to specific sites [133,134]. CD44, a PCSC marker, has been identified as a Hyaluronic acid receptor that activates the EMT pathway and promotes pancreatic cancer metastasis [134]. Tenascin-C enhances the expression of stem cell signaling components musashi homolog 1 (MSI1) and leucine-rich repeat G protein-coupled receptor 5 (LGR5). These molecules are positive regulators of the Notch signaling and target genes of the Wnt pathway, respectively, and promote the formation and growth of metastases [133]. The ECM components and their biological and physical properties are involved in the regulation of basic functions of immune cells, such as activation, proliferation and migration. The previously described M2 macrophages engineered with CAFs can drive cancer cell invasion in a CCL18-dependent manner [135]. Cytotoxic T-lymphocytes (CTLs) physiologically play an important role in eliminating cancer cells, but studies have found that they are often trapped in dense ECM compartments. Immune cells that are initially attracted to the tumor site by chemokines are prevented from migrating to the tumor core following contact with the area of increased stiffness [66,136]. Continuous ECM remodeling and overexpression of certain matrix components also promoted the recruitment of bone marrow cells, which are ultimately polarized to support ECM remodeling, CTL inhibition, tumor proliferation, and invasion [137,138]. Analysis of T-cell trajectories in tumors revealed that both CD4+ and CD8+ T-cells were trapped in fibronectin- and collagen-rich stromal regions, suggesting that the ECM may facilitate immune evasion by hindering proper infiltration of T-cells into tumors [66]. In addition, studies have speculated that laminin can induce a similar mechanism of dendritic cell tolerance and promote tumor immune escape [139]. Proteoglycan and Hyaluronic acid from ECM components can bind Toll-like receptors-2 and -4 to induce inflammatory gene expression, thereby exacerbating tumor-site inflammation [140,141]. Subsequently, cytokines and other inflammatory mediators that are secreted by tumor-associated immunosuppressive cells contribute to the stemness, tumorigeniccity, and metastatic potential of CSCs [142]. It has been confirmed that CSCs create a microenvironment to promote immunosuppression and tumor metastasis by secreting signals of tumor-specific CTL function and overexpression of these matrix components [143].

The process of CSC migration involves ECM degradation, remodeling of cell–cell and cell–ECM interactions, formation of adhesive plaques and invasiveness, and EMT transformation, which requires a lot of cellular energy [144]. The glycolysis dependence of CSCs can drive the degradation of the ECM, the formation of invasive structures, and cellular protrusions, leading to the migration and invasion of cancer cells [145]. Interestingly, the release of lactate, the final product of this process, alters the extracellular pH and promotes ECM degradation, leading to cancer cell migration [146].

In addition, massive ECM deposition, mainly collagen and hyaluronic acid, resulted in increased tumor stiffness in PDAC. A stiffer ECM induces the production of fibronectin, which binds extracellular collagen, fibrin, and heparan sulfate proteoglycans on one side and integrins on the other. An ECM with increased stiffness can enhance cell adhesion to the ECM, connect the ECM to the cytoskeleton through local adhesion proteins, and increase cytoskeletal tension through Rho/ROCK signaling activation [147]. Next, integrin aggregation initiates the recruitment of focal adhesion signaling molecules, such as FAK, Src, paxillin, and Rac, Rho, and Ras, which promote tumor progression [148]. Moreover, ECM stiffening can directly enhance PI3K activity and tumor invasiveness [149]. Surprisingly, increased matrix stiffness leads to elevated ROCK activity in PDAC with mutant SMAD4. These responses in turn stimulate ECM production, assembly of focal adhesions and signaling, and activators of transcription-3 (STAT-3) signaling that drive tumor progression [150]. Matrix stiffening can also induce EMT, which, in addition to leading to the acquisition of a more aggressive phenotype, also facilitates the transformation of cancer cells into stem cells that are more favorable for migration and invasion [151,152].

The YAP/TAZ transcriptional coactivator is a core component of the Hippo pathway and a sensor of the structural and mechanical characteristics of the cellular microenvironment [153]. Increased matrix stiffness in PDAC leads to YAP/TAZ activation, which in turn promotes the production of profibrotic mediators and ECM proteins. Activated YAP/TAZ can enhance tumor proliferation and survival by transactivating target genes that are related to cell cycle progression and anti-apoptosis and can also induce the expression of the proto-oncogene c-Myc to promote cell cycle progression [154,155]. More importantly, YAP/TAZ is closely related to ZEB1/2 and Twist, both of which can be used to regulate EMT, thereby inducing malignant features of cancer cells and CSC-related properties, such as tumor initiation, drug resistance, and metastasis [156]. Furthermore, YAP-mediated activation of myosin light chain 2 is critical for the generation of CAFs, contributing to matrix remodeling and tumor invasion [157]. Table 1 lists the above-mentioned mechanisms that are related to ECM and PCSC-induced metastasis.

## 6. ECM, PCSCs, and Chemoresistance

**Figure 1:**
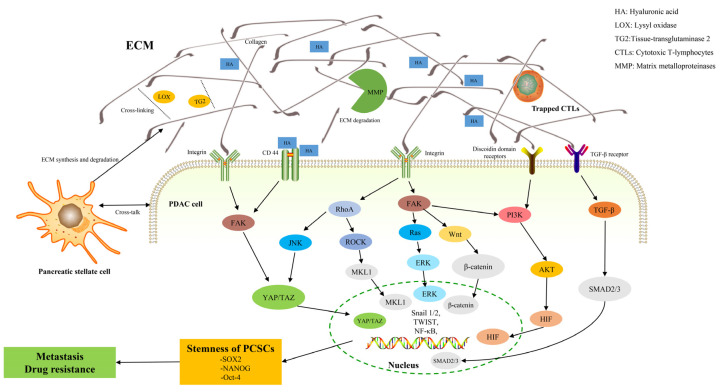
Signaling pathways that are associated with matrix stiffness. Matrix stiffness activates a large number of mechano-responsive signaling pathways in different cells through integrins, ion channels, and other transmembrane proteins. Pathways such as PI3K, RhoA-ROCK, and YAP/TAZ play major roles in this conduction. The central players in these signaling pathways can be connected to other molecules to eventually transform the changes in the ECM into related biological changes and phenotypes.

To date, the mechanisms of ECM-related chemoresistance have been identified, which can be roughly divided into two major categories, namely physical barriers (abnormal vascularization and matrix stiffness) and cell adhesion-related means (ECM tissue, mechanical signaling pathways, and pro-survival signaling pathways) [158] (Figure 1). Aberrant ECM remodeling results in increased matrix stiffness, vessel collapse, and reduced blood flow, which greatly reduces the ability of drugs to enter the tumor [159]. As previously described, dense fibrosis and abnormal vascularization in PDAC contribute to the formation of a hypoxic and pH-abnormal tumor microenvironment. Hypoxia also affects drug movement from the bloodstream to the tumor microenvironment, specifically affecting the activity of drug transporters and the expression and activity of phase I drug metabolizing enzymes [160,161,162]. Glycolysis under hypoxia results in the production of large amounts of lactic acid, which reduces the extracellular pH. The ability of the drug to cross the hydrophobic membrane is greatly reduced when the drug is electrically charged in an acidic environment [160]. In addition to the dense fibrotic ECM that impairs the ability of drugs to spread from blood vessels to cancer cells, most ECM proteins contribute to chemoresistance by activating EMT and oncogenic signaling pathways, including MAPK, PI3K, and YAP [163,164,165,166]. CSCs are also an important factor leading to chemotherapy resistance, but there are only few studies that are available on the relationship between the ECM and PCSC in chemoresistance. Therefore, this part focuses on the research progress of ECM-PCSC interactions and chemoresistance. Table 2 lists the mechanisms of action of the ECM in relation to chemotherapy resistance in this paragraph.

Recently, the physical properties of ECM and the role of its direct or indirect signaling pathways in the survival and maintenance of CSCs have become increasingly apparent [167]. In addition, to provide anchoring, the ECM receptor of CSCs mediates paracrine signaling that is involved in self-renewal and differentiation processes [168]. As previously described, hyaluronic acid-CD44 interactions increase the stemness (including NANOG and SOX2) and expression of drug resistance factors (MDR1) of PCSCs. Hyaluronic acid synthase 1–3 (HAS1-3) is a key enzyme in Hyaluronic acid synthesis, and its expression level is closely related to the prognosis of patients. 4-Methylumbelliferone inhibits Hyaluronic acid synthesis and is itself an approved drug for bile treatment [136]. Strikingly, Hyaluronic acid accumulation was significantly reduced in mouse models of pancreatic cancer that were treated with 4-MU [169]. Furthermore, other in vivo studies using a murine PDAC model demonstrated that treatment with pegylated human recombinant PH20-Hyaluronidase reduced Hyaluronic acid levels and improved gemcitabine therapy [15].

Cytotoxic agents have been the cornerstone of chemotherapy for PDAC, but more studies are needed to understand their effects on ECM dynamics and CSCs-acquired chemotherapy resistance. Recent studies have shown that the abnormal expression and activation of the transcription factor nuclear factor-erythroid 2-related factor 2 (NRF2) and its major negative regulator Kelch-like ECH-associated protein 1 (Keap1) are observed at different stages of PDAC, and are involved in tumor development, metastasis, and drug resistance [170,171]. During PDAC progression and metastasis, Keap1 is frequently mutated and silenced, resulting in abnormal stabilization of NRF2 [171]. NRF2 can be activated by stromal-derived factor 1α (SDF-1α) and IL-6, that are secreted by PSCs [172]. The activation of NRF2 can induce drug resistance of CSCs through the up-regulation of glutathione pathway. The activation of the glutathione pathway involves binding of TGF-β to its membrane receptor, followed by upregulation of P21. CSCs cell cycles that respond to TGF-β are slowed-down or stopped, which contributes to chemotherapy resistance because cytotoxic chemotherapy induces apoptosis by causing DNA damage exclusively in rapidly dividing cells [173,174]. In addition, the Keap1-NRF2 pathway is also involved in the chemotherapy resistance of PDAC by regulating the expression of drug resistance-related genes and cytoprotective antioxidant genes [175]. The tumor microenvironment promotes chemoresistance by maintaining the phenotype of CSCs, for example, collagen promotes CSC self-renewal through integrin signaling. The JNK signaling has been shown to promote the up-regulation of ECM-related genes. Thus, the JNK signaling may further lead to chemoresistance by establishing and regulating the CSC niche [176].

## 7. Pharmacological Targeting of ECM

CSCs rely on the favorable environment that is created by the ECM to obtain the necessary support for its development and survival. Thus, targeted therapy for the ECM may inhibit the growth of CSCs by disrupting its niche, and ultimately improving patient survival [177]. Since Hyaluronic acid leads to both increased intra-tumoral pressure that affects drug delivery and the acquisition of PCSC characteristics, it was hypothesized that targeting Hyaluronic acid would improve the efficacy of chemotherapy and patient prognosis [169,178]. The effect of PEGylated human recombinant PH20-Hyaluronidase (PEGPH20) was evaluated in combination with mFOLFIRINOX and albumin-bound paclitaxel plus gemcitabine in SWOG S1313 and HALO trials, respectively [179,180].

Collagen is one of the most basic components in the ECM, and it is also one of the ideal therapeutic directions. TGF-β plays a key role in collagen synthesis, so TGF-β signaling is the most promising target for inhibiting collagen synthesis. In animal models of PDAC, an anticoccidial named halofuginone has been shown to reduce collagen synthesis by inhibiting TGF-β signaling [181]. TGF-β is usually overexpressed in PDAC and blocking TGF-β-mediated signaling may enhance antitumor effects [173,174]. A clinical trial aiming to target TGF-β in PDAC is investigating the antitumor activity of SAR439459, a pan-TGF-β neutralizing antibody [182]. Vitamin D may also block collagen secretion by disrupting the TGF-β signaling pathway, helping to prevent tumor metastasis and enhance drug responses [183,184]. The inhibition of collagen cross-linking is also a therapeutic strategy to target ECM stiffness in cancer. In 2017, a randomized Phase II study of Simtuzumab, a humanized IgG4 monoclonal antibody that inhibits extracellular LOXL2, was performed in combination with gemcitabine in patients that were suffering from metastatic PDAC [132]. However, the results showed that a combination of gemcitabine and Simtuzumab did not improve clinical outcomes. Therefore, the complex tumor-stromal interaction of LOXL2 in PDAC tumorigenesis requires further investigation and re-evaluation of the efficacy of using anti-LOXL2 antibodies or small molecule LOXL2 inhibitors in the treatment landscape of metastatic PDAC [185].

Considering the importance of CD44-hyaluronic acid interactions in tumor cells, they may be promising therapeutic targets for cancer therapy. Several research groups are working to evaluate the antitumor effects of CD44 antibodies, such as bivacizumab, the first humanized monoclonal antibody against CD44v6 [186]. Now, more CD44 antibodies are entering clinical trials, such as the RO5429083 trial in solid tumors. Research on the application of fibronectin in cancer therapy has mainly focused on its application as a target for precision drug delivery. Several targeted therapies targeting the extra domain B of fibronectin have been developed in tumors such as bowel cancer and skin cancer [187]. But therapeutic trials and data for pancreatic cancer are currently lacking.

The inhibition of ECM production by modulating the activation state of PSCs is considered a viable therapeutic strategy. The transcriptional regulation of CAFs and PSCs is regulated by vitamin D receptors (VDR), so the treatment with Paricalcitol can reprogram the matrix, reduce inflammation, and improve the response to gemcitabine [188,189]. Vitamin D receptor agonists restore PSCs to a quiescent state, thereby reducing tumor fibrosis and enhancing chemotherapeutic drug delivery. Likewise, PSCs also express retinoic acid receptors, which interact with the vitamin A metabolite all-trans retinoic acid (ATRA) [190]. By binding to retinoic acid receptor β, ATRA reduces PSC activation, inhibits ECM remodeling, and also attenuates the ability of PSCs to sense external mechanical signals from a stiff ECM [190]. The composition of the ECM can be further altered by inhibiting angiotensin, a profibrotic cytokine. Losartan, an angiotensin receptor blocker, was found to reduce the expression of TGF-β, hyaluronan synthase 1–3, and collagen I in cancer-associated fibroblasts [191,192]. Enalapril, another renin-angiotensin system inhibitor, has been shown to inhibit the progression of PDAC in combination with aspirin [193].

There are other therapeutic ideas aiming to target the MMP to alter ECM stiffness. For example, MMP-9 promotes aggressive and metastatic phenotypes in tumor cells, and its overexpression increases the aggressiveness of cancer cell lines in vitro [194]. Targeting integrin-mediated signaling is another strategy for ECM therapy. In theory, it is possible to disrupt signals from the extracellular or intracellular environment by using integrin inhibitors to disrupt ECM mechanical sensing [177]. Targeted glutamine metabolism and hexosamine biosynthesis pathways has been shown to have profound effects on the ECM and CSC self-renewal of PDAC among metabolic inhibitors [195]. However, due to the specific environmental effects of these factors and the complex interaction between the ECM and tumor cells, the current treatment for these regimens is not beneficial. Thus, a detailed understanding of the interactions between ECM proteins, glycans, cancer-associated fibroblasts, and cancer cells will ultimately lead to more effective treatment strategies of cancer. An overview of ECM targeting inhibitors and monoclonal antibodies that are currently under investigation in clinical trials is shown in Table 3. In addition, targeting related genes and signal transduction that is regulated by the ECM is also one of the therapeutic directions. JNK signaling promotes the up-regulation of ECM-related genes and CSC self-renewal. JNK signaling is involved in regulating the niche that allows CSCs to evade chemotherapy and promote metastasis in mouse models. Treatment with JNK inhibitors reduces ECM protein expression and potentiates the chemotherapy effect [176]. The STAT3 inhibitor BBI-608 (Napabucasin) in combination with albumin-bound paclitaxel and gemcitabine is currently being tested in a Phase III clinical trial in patients with metastatic PDAC [196]. The interaction of CSCs with the tumor microenvironment also regulates the plasticity and function of CSCs, which contributes to intra-tumor heterogeneity [197]. Treatment with Sonic hedgehog (Shh) inhibitors improved tumor microvascular density and survival in mouse models [198]. However, in clinical trials, Shh inhibitors in combination with chemotherapy failed to improve the overall survival and even resulted in worse outcomes [199,200]. Recent studies suggested that this is a consequence of the heterogeneity of CAFs in tumors. Blocking the Shh signaling pathway may lead to a transformation of myofibroblast-CAF into inflammatory-CAF, promoting an immunosuppressive tumor microenvironment [201]. Therefore, a deeper understanding of the biology and function of different CAF subtypes is beneficial to improve the therapeutic effects.

Clinical trials involving different inhibitors are being conducted in different clinical settings, primarily for ECM-induced stemness of PCSCs. For example, the inhibitors of Rho-associated kinase (ROCK) alter the contractility of PDAC cytoskeleton and CAF, may benefit drug delivery and inhibit metastasis. ECM disorder occurrence and PDAC migration and invasion are prevented when ROCK is suppressed. Other mouse models showed that a pre-chemotherapy administration of ROCK inhibitors increased the chemotherapy response of the primary tumor and helped to prevent the development of liver metastases [202,203,204]. T13148 was the first ROCK inhibitor that was investigated for the treatment of solid tumors, but further development of this compound is advised due to drug side effects [205].

## 8. Conclusions

The ECM provides a favorable environment for CSCs to survive and interact with each other, which play an important role in metastasis formation of PDAC and chemotherapy resistance. Preclinical studies suggest that a treatment aiming to target the dense fibroproliferation-promoting ECM proteins may provide promising new therapeutic options for PDAC patients. However, ECM-targeted therapy still needs more in-depth molecular characterization and discussion according to the clinical practice results. We need to dissect the role of abnormal ECM dynamics in re-shaping the tumor microenvironment to develop more feasible therapeutic strategies for deadly cancers such as PDAC.

## Figures and Tables

**Table 1:** Mechanisms of metastasis in PDAC by the action of ECM and PCSCs.

ECM-Related Components	Target of Action	Mechanism	Effect
Collagen	LOXs	ECM remodeling/EMT	Metastasis/stemness
Elastin			
Hyaluronic acid	CD44	EMT	Metastasis
	Toll-like receptors-2/-4	Immune evasion	Metastasis/stemness
Tenascin-C	MSI1/LGR5	Notch/Wnt pathway	Metastasis
CAFs	M2 macrophages	CCL18-dependent manner	Metastasis
Fibronectin	CD4+/CD8+ T-cells	Immune evasion	Metastasis
Collagen			
Laminin	Dendritic cell	Immune evasion	Metastasis
ECM-cell interaction	Glycolysis dependence of CSCs	Degradation of ECM	Metastasis
Matrix stiffness		Rho/ROCK pathway	Metastasis
		PI3K pathway	Metastasis
		EMT	Metastasis/stemness
		YAP/TAZ	Metastasis/stemness

**Table 2:** Mechanisms of chemoresistance in PDAC by the action of ECM and PCSCs.

ECM-Related Factors	Point of Action	Mechanism
Abnormal vascularization/high fibrosis	Hypoxia	Affecting affect drug transport
	pH	
	Matrix stiffness	
ECM proteins		EMT
		MAPK signaling pathway
		PI3K signaling pathway
		YAP signaling pathway
ECM-PSCS interaction	HA-CD44	Increase the stemness and MDR1
	Keap1-NRF2	Up-regulation of glutathione pathway
		Regulating the expression of drug resistance-related genes
	JNK	Up-regulation of ECM related genes

**Table 3:** Overview of clinical trials targeting the ECM in PDAC.

Drug Name	Mechanism	Clinical Trial Phase	NCT Registry Number
PEGPH20	Degradant of Hyaluronan	II	NCT01839487
		Ib/II	NCT01959139
		III	NCT02715804
		Ib/II	NCT03193190
Hydroxychloroquine (HCQ)	Inhibition of JNK-related autophagy	I/II	NCT01506973
GDC-0449	Inhibitor of Hedgehog	I	NCT00878163
		II	NCT01088815
		II	NCT01195415
		I/II	NCT01064622
		II	NCT01088815
IPI-926	Inhibitor of Hedgehog	I	NCT01383538
		Ib/II	NCT01130142
AT13148	Inhibitor of ROCK and AKT kinases	I	NCT01585701
Paricalcitol	Vitamin D receptor	II	NCT03520790
	Disrupting the TGF-β signaling pathway	II	NCT03415854
ATRA	Inhibitor of PSCs activation	I	NCT03307148
Losartan	Inhibition of angiotensin	I	NCT01276613
		II	NCT01821729
RO5429083	CD44 antibody	I	NCT01358903
SAR439459	Pan-TGF-β neutralizing antibody	I	NCT03192345
Napabucasin	Inhibitor of STAT3	III	NCT02993731
Simtuzumab	Inhibitor of LOXL-2	II	NCT01472198
BT1718	Inhibitor of MT1-MMP	I/IIa	NCT03486730
Volociximab	Inhibitor of Integrin	II	NCT00401570
